# *I just lost it!* Fear and anger reduce the sense of agency: a study using intentional binding

**DOI:** 10.1007/s00221-018-5461-6

**Published:** 2019-03-02

**Authors:** Julia F. Christensen, S. Di Costa, B. Beck, P. Haggard

**Affiliations:** 1 Institute of Cognitive Neuroscience, University College London (UCL), Alexandra House, 17 Queen Square, London, WC1N 3AR UK; 2 Present Address: School of Psychology, University of Kent, Canterbury, UK

**Keywords:** Sense of agency, Intentional binding, Fear, Anger, Loss of control

## Abstract

Two recent studies have demonstrated that increases in arousal states lead to an increase people’s sense of agency, i.e., the subjective experience of controlling one’s own voluntary actions (Minohara et al. in Front Psychol 7:1165, 2016; Wen et al. in Conscious Cogn 36:87–95, 2015). We here extend these findings by showing that arousal states with negative emotional valence, such as fear and anger, *decrease* sense of agency. Anger and fear are negative emotional states. Anecdotally, they are often invoked as reasons for losing control, and neuroscientific evidence confirms important effects on the brain’s action control systems. Surprisingly, the subjective experience of acting in anger or fear has scarcely been investigated experimentally. Thus, the legal notion that these intense emotions may undermine normal voluntary control over actions and outcomes (the ‘Loss of Control’, a partial defence for murder) lacks any clear evidence base. In three laboratory experiments, we measured sense of agency using an implicit measure based on time perception (the “intentional binding” paradigm). These actions occurred in either an emotionally neutral condition, or in a fearful (experiments 1 and 2) or angry state (experiment 3). In line with our hypotheses, fear or anger reduced the subjective sense of control over an action outcome, even though the objective causal link between action and outcome remained the same. This gap between the objective facts of agency, and a reduced subjective experience of agency under emotional conditions, has important implications for society and law.

**Electronic supplementary material:**

The online version of this article (10.1007/s00221-018-5461-6) contains supplementary material, which is available to authorized users.

## Introduction

‘Sense of agency’ refers to the subjective experience of controlling one’s own voluntary actions, and, through them, of causing events in the external world. [for a thorough review of sense of agency research, please see (David et al. 2008; Haggard 2017)]. Two recent studies have demonstrated that unspecific arousal states (induced by colours or physical effort) increase people’s sense of agency over their actions (Minohara et al. 2016; Wen et al. 2015), while factors that decrease sense of agency will tend to reduce feelings of control and responsibility (Yoshie and Haggard 2013, 2017).

Being in control of, and thereby being responsible for one’s actions, is a key concept of criminal law. Although fear and anger are sometimes offered as reasons for reduced responsibility over one’s own actions, the effects of negatively valenced arousal states on sense of agency remain unclear. Extreme stress and negative emotional states influence brain mechanisms underlying action control, focussing cognition on a single action, and limiting the consideration of alternative responses and their outcomes (Easterbrook 1959). Thus, one might predict that induction of a negative emotional state such as fear or anger would reduce the sense of control over one’s actions. Indeed, several studies have shown that negative action outcomes, such as fearful or angry human vocalisations produced by voluntary key presses, reduce sense of agency over the committed action (Barlas et al. 2018; Christensen et al. 2016; Gentsch et al. 2015; Takahata et al. 2012; Yoshie and Haggard 2013, 2017; but see; Moreton et al. 2017, for contrary findings). Other studies have demonstrated that positive emotion inductions enhances sense of agency (Aarts et al. 2012), and alters awareness of the intention to act (Rigoni et al. 2015). However, none of those studies reproduces the scenario behind a *Loss of Control* legal defence, in which the key factor is the defendant’s emotional state prior to and during the action, rather than the emotional quality of the outcome. Rigoni et al. (2015) found increased awareness of intention to act when participants were in a positive emotional state, but found no effects of negative emotional states. These authors used a combined emotion induction procedure; participants listened to music of different emotional valence, while they read matching emotion-inducing sentences (positive, neutral, negative). The authors discuss whether their lack of findings for the negative emotion condition might be due to a weaker emotion induction effect for negative emotions.

“*I just lost it!*” is a common phrase in court rooms the world over. Furthermore, *Loss of Control* is a partial defence for murder in many jurisdictions. In English Law, for example, a Defendant will not be convicted of murder if the “*killing resulted from [the Defendant’s] Loss of Control*” (Coroners and Justice Act 2009, article 54, sect. 1a), as long as a range of conditions are met. These conditions include the emotional state of the defendant. This paper tests whether scientific evidence from psychological laboratory experiments supports the link between emotion and action control implied by the Loss of Control defence. The law cannot rely only on a defendant’s subjective reports that they lost control because of possible abuse of this defence for secondary gain and evasion of punishment. Rather, the law must primarily consider objective facts about whether the agent could have controlled their actions, while at the same time being aware of any possible biases in the agent’s reported subjective experience. Thus, the law must, directly or indirectly, confront one of the major questions of cognitive neuroscience of agency: how do people *experience* their control over their voluntary actions? In normal circumstances, the objective physiological facts of motor action largely overlap with subjective experience. Agents are normally aware of what they are doing. However, research in cognitive neuroscience of voluntary motor control has shown that the usual link between the objective facts of control and the subjective feeling of control over a motor action can be altered under certain conditions. Thus, objective agency is expressed by the fact that “Agent A does Action B”. Subjective sense of agency is A’s feeling that they are doing/have done B (Desantis et al. 2011; Hommel 2015; Wegner et al. 2004). In healthy adult life, objective agency and subjective sense of agency are well aligned: we feel a strong sense of control over our own actions, and only over our own actions.

In cases of violent and aggressive actions, such as homicides, the emotional states of fear and/or anger may feature strongly. Both these emotions prepare the body physiologically for action. However, the phrase “*I just lost it*” suggests that agents experience a reduction in *subjective* sense of agency over actions committed in fearful and angry states. Thus, strong emotions might open a gap in the normal alignment between the subjective experience and objective facts of agency on which the law relies. In principle, scientific data on both the objective controllability of action under emotion and the subjective experience of agency should be highly relevant to this question, but, in practice, current law has not been informed by the evidence base of cognitive or brain sciences. Previous studies reported reduced sense of agency over actions that produced negative, compared to either positive or neutral, outcomes (Barlas et al. 2018; Christensen et al. 2016; Gentsch et al. 2015; Yoshie and Haggard 2017). One might suggest, therefore, that negative emotions might cause a reduction in the sense of control over one’s own actions and their external outcomes. However, no study has yet investigated how the subjective sense of agency might be altered by inducing states of fear or anger.

Despite the negative valence of both fear and anger states, there are some reasons to predict that they might have different effects on sense of agency. Fear depends on subcortical circuits which operate preconsciously—notably the amygdala (LeDoux 2003)—while the anger state recruits a broader cortical network (Denson et al. 2009). Moreover, fear and anger are associated with different action tendencies; fear facilitates automatic withdrawal responses or action inhibition (e.g., fleeing or freezing), at least as an initial effect of fear. In contrast, anger facilitates approach behaviours (e.g., aggression, fighting; Carver and Harmon-Jones 2009; Davidson 1992; Frijda 1987). Here, we have focussed on how these emotional states influence the experience of a voluntary instrumental action, rather than the actual flight/fight behaviours with which they are associated. On this basis, we hypothesised that fear states would abolish sense of agency over outcomes of voluntary action, for two reasons. First, fear is associated with preconscious, automated behavioural patterns (LeDoux 2003) and might thus have decreasing effects on sense of agency, making voluntary actions feel involuntary. Second, flight responses triggered by fear involve abandoning instrumental agency over the current environment, again abandoning voluntary control, in favour of moving to a different, safer environment.

Regarding the anger state, one prediction would be that anger states enhance sense of agency. Certainly, motivation of goal-directed actions can be boosted by anger [though Aarts et al. (2010) only found this effect when the outcome was rewarding), and anger is subjectively experienced as a hyperkinetic phenomenon (“I knew I shouldn’t have hit him, but I was so angry…”]. On the other hand, it remains unclear whether the basic, impulsive motor actions that are associated with the anger state indeed produce a genuine sense of agency in the same way as reason-responsive, goal-directed actions (“… that I suddenly found myself punching him in the face”). In that case, anger states might conceivably be associated with reduced sense of agency.

Three experiments tested these predictions. Established laboratory models of fear (experiments 1 and 2) and anger (experiment 3) were induced in healthy volunteers, who made voluntary keypress ‘actions’ that caused a tone (i.e., the ‘outcome of the action’) 250 ms later. We used this well-known ‘intentional binding’ paradigm (Haggard et al. 2002), to obtain an implicit measure of sense of agency. This allowed us to investigate how fear and anger states influence sense of agency over actions.

**Fig. 1:**
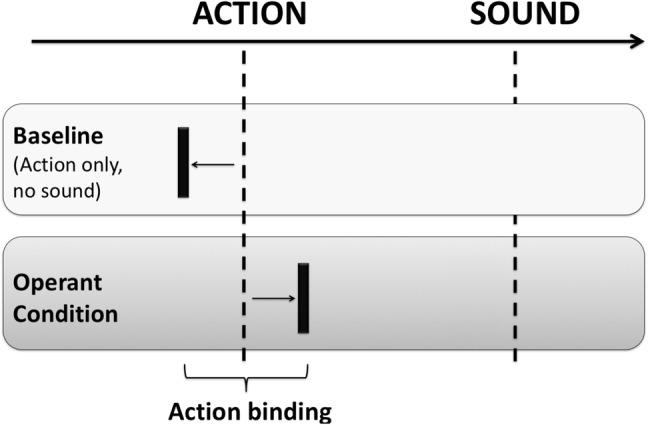
Schematic of action binding. Action binding is a measure of the subjective experience of the linkage between an action and its outcome (Haggard et al. 2002). Action binding is calculated as the difference in the perceived time of the action between the operant condition, in which the action produces an auditory tone, and a baseline block, in which the action does not produce any tone. The perceived time of a voluntary action shifts towards the time of a subsequent tone in the operant condition, relative to the baseline

We specifically focussed on action binding, defined as the shift in the perceived time of an action (keypress) towards the outcome (tone) it produces. Previous research suggests that action binding is specific to conditions, where an action is internally generated and executed voluntarily (Borhani et al. 2017). Therefore, action binding provides a direct measure of the degree to which the mental representation of an action is linked to the action’s outcome: this laboratory measure seems to capture the essential cognitive requirement of legal notions of responsibility, namely, to be aware of and in control of one’s own actions (Fig. 1).

## Methods

### Participants

A sample size of 20 participants per experiment was determined with *GPower* 3.1 (Faul et al. 2007) using the a priori procedure for within-subject *t* tests (assuming a large effect size of 0.80; alpha = 0.05; power = 0.95; Cohen 1988). In total, 60 female right-handed volunteers (mean age = 23.53, SD 4.01) participated in the study (time reimbursement: £7.50/h). Ethical approval for all studies was obtained from the UCL Research Ethics Committee (project code 4435/001), prior to commencement of any testing activities. Participants gave written informed consent before taking part in the study.

### Materials and procedure

#### Intentional binding paradigm

The intentional binding paradigm is a quantitative proxy measure of sense of agency that has been extensively used. Our procedure followed that of the previous studies (Haggard et al. 2002; see also supplementary material). Briefly, in this task, on each trial, participants are instructed to press a key on the keyboard in front of them at a time of their choosing, while fixating the centre of a clock displayed on the screen with a continuously rotating clock hand. A tone occurs at a fixed duration of 250 ms after each keypress. This brief interval between action and tone gives participants the impression of causing the tone. Participants are then prompted to say, where the clock hand was on the clock face in the moment they pressed the key. The experimenter records their verbal response, and launches the next trial. The judgement error between the reported and actual time of action is calculated. Next, action binding is obtained by calculating the difference in average judgement error for actions made in a baseline block, in which no tone occurs, and actions made in an operant condition, where the action always elicits the tone 250 ms later. Action binding serves as an implicit marker of sense of agency.

Experiments 1 and 2 combined a fear induction procedure with the intentional binding task (see “Fear induction”). Experiment 3 combined an anger induction procedure with the intentional binding task (see “Anger induction”). In all three experiments, participants completed six time estimation blocks of 32 trials each. Blocks 1 and 6 were baseline blocks (i.e., no tone occurred). Blocks 2–5 were operant blocks (i.e., a tone occurred 250 ms after the participant’s keypress).

#### Fear induction

The Threat of Shock paradigm was used to induce fear (Davis et al. 2010; Robinson et al. 2013; Schmitz and Grillon 2012). In this procedure, some moderately painful shocks are delivered early on in the experiment. This causes participants to anticipate more shocks, even on trials, where they actually receive no shock. Thus, interest focusses on how participants’ expectation, or fear, of subsequent shocks alters cognition—in our case, sense of agency.

To deliver the painful stimulation, a Digitimer DS7A constant current stimulator was used. Two electrodes were placed on the back of the participants’ left hand, since they were using the right hand for the keypress (all participants were right-handed). For the pain calibration, prior to experiments 1 and 2, each participant’s individual pain threshold was determined using a stepwise approximation procedure, increasing the stimulation in small steps of 1 mA, until the shocks were “painful but definitely bearable” (see supplementary materials for further details).

Our participants performed 4 operant blocks of which 2 blocks were “threat” blocks (the word “threat” was displayed on the screen) and participants were informed they could receive a shock at any time. The other 2 blocks were “safe” blocks (the word “safe” was displayed on the screen) and participants were informed that they would receive no painful shocks. Threat and Safe blocks were alternated, and the starting block was counterbalanced (experiment 1), or interleaved in a fixed order (threat–safe–threat–safe; experiment 2). In experiment 1, the electric shocks occurred simultaneously with the keypress. In experiment 2, they occurred at the time of the subsequent tone. This latter arrangement controls for potential effects of prior entry (subjective experience of salient events occurring earlier in time; Spence and Parise 2010).

**Fig. 2:**
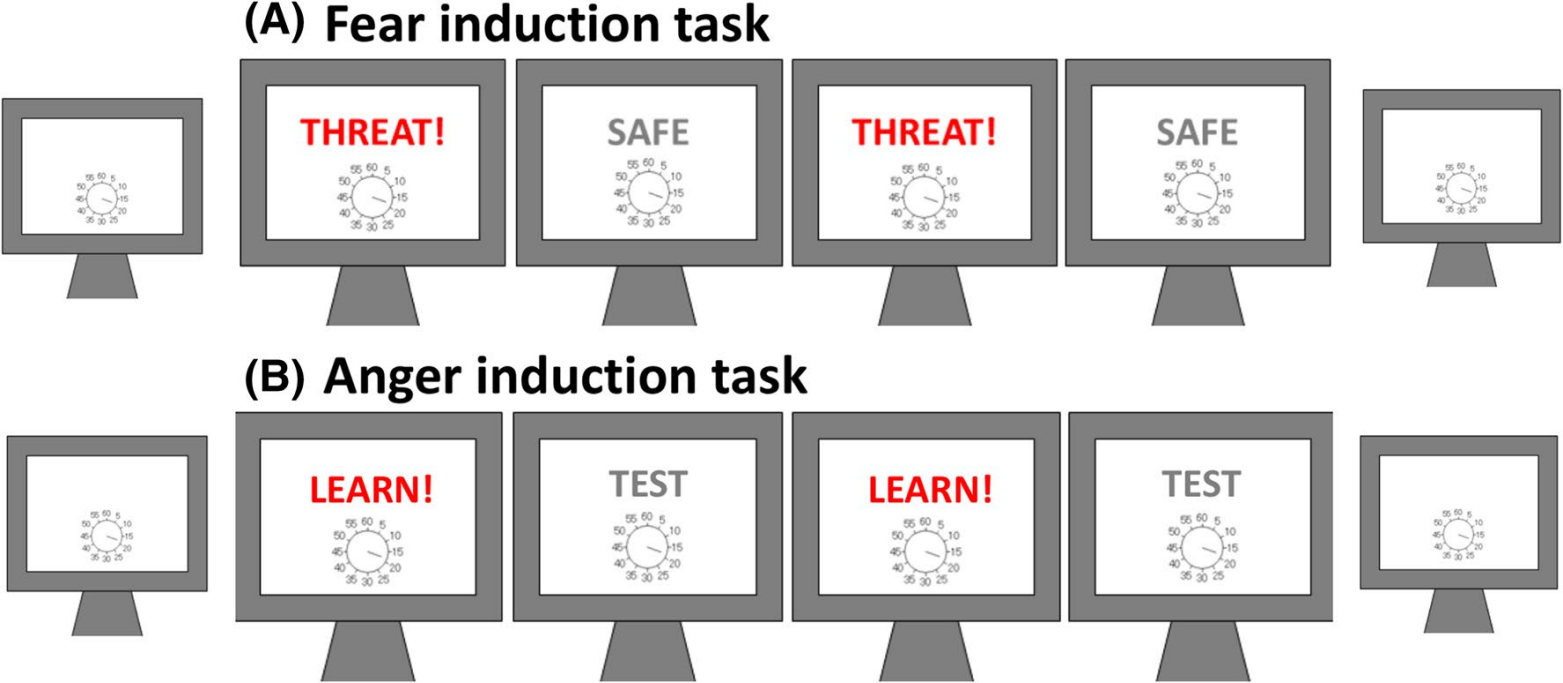
**A** Illustration of fear induction procedure. Threat and Safe blocks were alternated, and the starting block was counterbalanced (experiment 1), or interleaved in a fixed order, always A–B–A–B (threat–safe–threat–safe; experiment 2). **B** Illustration of anger induction procedure. The operant blocks were interleaved in a fixed order, always A–B–A–B (learn–test–learn–test; experiment 3). In all three experiments, participants performed 2 baseline blocks, one before and one after the 4 operant blocks. See text and supplementary material for full details

Our implementation of this paradigm follows the procedure as detailed below and is explained further in the supplementary material. Participants in the “fear” action binding paradigm were informed that occasional randomly-interleaved shocks to their hand might occur in some blocks of the experiment. For this, blocks of trials in the intentional binding task were labelled on-screen as “threat” or “safe”. Participants were informed that they might receive a painful shock at any time in “threat” blocks. Five painful shocks were given on random trials early in “threat” blocks. Expectation (or fear) of painful shock in threat blocks was hypothesised to influence action binding. In “safe” blocks, participants were informed they would not receive any painful shocks, and accordingly, no painful shocks were given (experiment 1), or five early non-painful shocks at detection threshold were given (experiment 2, see supplementary methods for justification). Participants also performed 2 baseline blocks, one before and one after the 4 operant blocks. Shock trials were discarded, and action binding was analysed for trials without shocks (Fig. 2).

#### Anger induction

In experiment 3, we used a frustration paradigm called the “Impossible Task” which is a common procedure for inducing anger or frustration (Buss 1961; Taylor 1967). The rationale behind this paradigm is that the participant is assigned a task, but successful performance on the assignment is never possible, no matter how hard the participant tries. This task has been used to provoke anger states, since the participant acts in expectation of a reward, but never obtains it (Blair 2012).

For the present experiment, participants were informed that in addition to the Clock estimation task, a reaction time (RT) task would be embedded in the 4 operant blocks. Our implementation of this paradigm follows the procedure as detailed below and is explained further in the supplementary material. Participants were instructed that whenever the screen background colour would change, they should press F1 as quickly as possible using their right hand, reaching across the body midline horizontally. They were told that in 2 “learning” blocks they would receive feedback on their performance. They were promised a reward of £2.50 in addition to their final pay if they were fast enough in the RT trials. If they heard a “beep” sound, that would mean that they had been fast enough. If they heard a “buzz” sound, that would mean they had not been fast enough, and they had lost 25p of the £2.50. In fact, the program was set such that the outcome tone was always a buzz. Thus, feedback was misleading, and frustrating for participants, and the “learning” blocks in fact aimed to induce anger. In the 2 remaining operant blocks, the “test” blocks (control condition), participants were informed that they could ‘practice the task’ without affecting their potential reward bonus. They were told that their RT would be monitored as in the learning blocks and that they were to perform as the task as quickly as possible, just as they had learned in the “learning” blocks. Learning and test blocks were interleaved, in a fixed order, starting with a learning block. Probe reaching trials were discarded, and only action binding trials were analysed. Again, participants performed 2 baseline blocks in which they made judgements of actions in the absence of tones. The baseline blocks occurred both before and one after the 4 operant blocks (Table 1).

**Table 1:** Stimulus parameters, descriptive measures and post-session questionnaires for the three experiments

	Fear				Anger	
	Experiment 1		Experiment 2		Experiment 3	
	Mean	SD	Mean	SD	Mean	SD
Age	25.2	*4.67*	21.95	*3.33*	23.45	*3.39*
Detection threshold (µv)	25	*5.13*	20.75	*4.17*	NA	*NA*
Pain threshold (µv)	225.75	*118.15*	178.25	*88.20*	NA	*NA*
Intensity rating	7.35	*0.99*	6.1	*2.21*	NA	*NA*
Estimated number of shocks/probes (fear/anger blocks)	10.25	*3.20*	10.2	*3.41*	7.6	*2.30*
Estimated number shocks/probes (neutral blocks)	NA	*NA*	5.6	*4.84*	6.4	*2.35*
How scared?	1.15	*1.35*	0.8	*1.77*	− 2.1	*1.21*
How angry?	− 2.15	*1.18*	− 1.05	*1.23*	0.8	*1.61*
BAS drive	12.7	*2.23*	9.05	*2.14*	11.4	*1.75*
BAS fun seeking	12.9	*1.97*	8.6	*2.28*	12.32	*1.60*
BAS reward responsiveness	18.1	*1.23*	7.05	*1.82*	18.25	*1.29*
BIS	20.85	*4.43*	13.85	*2.39*	22.05	*2.84*

Threshold values refer to level of electrical stimulation just detectable, or just experienced as painful. Intensity rating is a subjective estimate of pain level at threshold. Number of shocks/probes: participant’s post-session estimate of number of shocks received in experiments 1 and 2, or of number of probe reaching trials in experiment 3

## Results

Manipulation checks showed that participants indeed experienced the emotional states targeted. In the post-session questionnaire of the fear experiment (experiment 2) participants reported to have been fearful during the threat blocks (*m* = 0.8, SD 1.77; range − 3 to + 3), and not fearful in the safe blocks (*m* = − 2.85; SD 0.49; range − 1 to − 3); (*t* = 2.09, *df* = 19, *p* < 0.001). Likewise, in the anger experiment (experiment 3), participants reported to have been angry/frustrated during the anger blocks (*m* = 0.8, SD 1.61; range − 3 to + 3) and less angry in the control blocks (*m* = 0.1; SD 1.65; range − 3 to + 3); (*t* = 2.09, *df* = 19, *p* = 0.006). See supplementary materials for details. Shock trials (experiments 1 and 2) and probe reaching trials (experiment 3) were discarded, and only action binding trials were analysed.

### Main analysis

For both experiments 1 and 2, separate 2 × 2 repeated measures (RM) ANOVAs were conducted with the factors Occurrence (1st time, 2nd time, to control for order effects) and State (Fear, Neutral). In experiment 1, ANOVA revealed a significant main effect of State (*F*(1,19) = 4.414, *p* = 0.049, $\eta _{{\text{p}}}^{2}$ = 0.189 90% confidence intervals [CI] for $\eta _{{\text{p}}}^{2}$ = [0.0004, 0.4089]). No other main effects or interactions were significant (Occurrence: *F*(1,19) = 0.353, *p* = 0.559, $\eta _{{\text{p}}}^{2}$ = 0.018; Occurrence × State: *F*(1,19) = 0.619, *p* = 0.441, $\eta _{{\text{p}}}^{2}$ = 0.032). In experiment 2 there was a trend of State (*F*(1,19) = 3.836, *p* = 0.065, $\eta _{{\text{p}}}^{2}$ = 0.168, 90% CI [0.0000, 0.3893]), in the same direction as experiment 1. Participants showed less action binding in a fear state. No other main effects or interactions were significant (Occurrence: *F*(1,19) = 0.310, *p* = 0.584, $\eta _{{\text{p}}}^{2}$ = 0.016; Occurrence × State: *F*(1,19) = 1.308, *p* = 0.267, $\eta _{{\text{p}}}^{2}$ = 0.064).

**Fig. 3:**
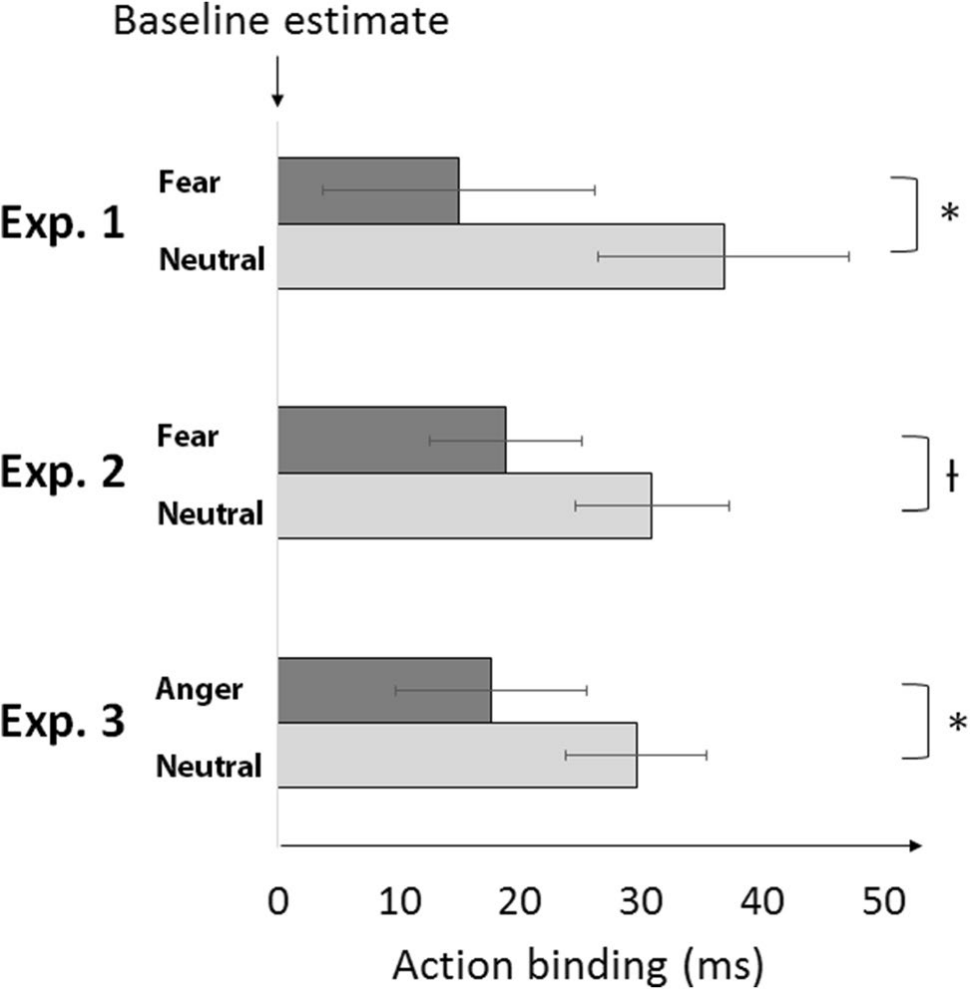
Action binding in emotionally neutral control blocks, and in conditions inducing fear or anger. Error bars represent SEM. *p < 0.05, *ƚ* = 0.065

For experiment 3, a 2 × 2 repeated measures (RM) ANOVA was conducted with the factors Occurrence (1st time, 2nd time) and State (Anger, Neutral). ANOVA revealed a significant main effect of State (*F*(1,19) = 4.847, *p* = 0.040, $\eta _{{\text{p}}}^{2}$ = 0.203, 90% CI = [0.0051, 0.4226]). Participants showed reduced action binding when angry. No other main effects or interactions were significant (Occurrence: *F*(1,19) = 0.385, *p* = 0.542, $\eta _{{\text{p}}}^{2}$ = 0.020; Occurrence × State: *F*(1,19) = 1.860, *p* = 0.189, $\eta _{{\text{p}}}^{2}$ = 0.089). See supplementary material for additional details (Fig. 3).

## Discussion

We found that both fear and anger inductions reduced our chosen implicit measure of sense of agency, namely intentional binding, or the perceptual attraction of a voluntary action towards its outcome. The prepotent action tendency in response to a fearful event is withdrawal, while for anger, it is approach (Carver and Harmon-Jones 2009; Davidson 1992; Frijda 1987; LeDoux 2003). However, these contrasting polarities of emotional modulation for fear and anger were not seen for our subjective sense of agency measure: both states resulted in a reduction of participants’ sense of agency.

Our result extends previous work in two important ways. First, our effect was found for *action binding*, while previous studies found valence effects primarily for *outcome* binding (Gentsch et al. 2015; Takahata et al. 2012; Yoshie and Haggard 2013, 2017). Action binding is potentially a more informative measure of action-outcome association than outcome binding: it is independent of the physical characteristics of the outcome event, while outcome binding is not (Wolpe and Rowe 2014). Second, we studied how an emotional *state* influenced sense of agency, rather than perception of events that were themselves emotionally significant. Again, this makes it unlikely that our time estimation results merely reflect specific features of our stimuli.

Fear and anger are both negative emotions, and both reduced our implicit measure of sense of agency. In that sense, we replicate previous findings of a reduced sense of agency in the presence of negative, compared to positive, emotion (Yoshie and Haggard 2013). Importantly, however, the negative emotion in our study was not linked to any specific event on those trials analysed for binding. Rather, negative emotion was linked to the participant’s emotional *state* at the time of acting. In both cases, the impact of negative emotion was a reduction in sense of agency. This could be interpreted as a psychological distancing from action outcomes.

Our study focussed on just two specific emotions that have been classically associated with loss of control, and potentially with reduced responsibility. Moreover, we found that both these emotions had comparable effects on our measure of sense of agency. Therefore, one might ask whether modulations of sense of agency are specific for particular emotions at all, or might alternatively reflect non-specific factors accompanying these emotions, such as general arousal. Many psychological studies distinguish emotional valence from arousal by demonstrating opposite directions for effects of positive and negative emotions. In contrast, arousal is generally assumed to be unipolar: both positive and negative emotions are thought to increase arousal (Lang and Bradley 2010; Lang et al. 2008). We found that two negative emotions both had effects in the same direction on sense of agency. Thus, arousal interpretations cannot be entirely ruled out. Nevertheless, we think that non-specific arousal cannot explain all emotional modulations of sense of agency for several reasons. First, the best-established effect of arousal on time perception is a speeding up of an ‘internal clock’, probably mediated by transient fluctuations in dopamine (for a review, see Droit-Volet and Meck 2007). However, those studies were based on changes in *duration* perception, rather than the cross-modal event perception studied here. Consistent shifts in perception of a single event, such as our action binding measure, are not easily explained by mere changes in arousal (Droit-Volet and Meck 2007). Second, authors of previous experimental studies have suggested that emotional stimuli that are equally arousing could have opposite effects on sense of agency, with the direction of the effect depending on their negative vs positive valence. In studies of financial wins and losses, or emotional sounds as action outcomes, negative outcomes reduced intentional binding relative to neutral outcomes. Yet enhancing effects of positive outcomes on intentional binding were small or absent, relative to neutral outcomes (Gentsch and Synofzik 2014; Takahata et al. 2012; Yoshie and Haggard 2013). Since participants in those studies rated positive and negative stimuli as equally arousing, arousal cannot readily explain such valence-dependent effects. Finally, two studies that specifically aimed to investigate effects of unspecific arousal on sense of agency both showed *stronger* sense of agency under high arousal conditions (Minohara et al. 2016; Wen et al. 2015)—opposite to the effects of fear and anger states that we found here (high arousal states of negative valence).

Our result should not be overinterpreted, either scientifically or normatively. Importantly, a reduced sense of agency does not imply that participants had *no* sense of agency, nor that they acted involuntarily. We assume that our participants retained full awareness of their actions and outcomes, and that their keypress actions were mediated by cortical voluntary motor systems throughout. Our findings merely suggest that they experienced less linkage between action and outcome under strong emotion. On the normative side, the fact that sense of agency is reduced by negative emotional states does not demonstrate total lack of responsibility, nor condone any specific action. *Feeling* less responsible does not necessarily *make* one actually less responsible (see supplementary material for a focussed discussion of relevance of our results to legal concepts of responsibility). For example, the law might reasonably require individuals to manage situations of high emotion so as to avoid irresponsible actions. Our surprising finding of anger-induced reduction in sense of agency may be particularly relevant in the context of anger management. The combination of increased drive to act, together with a reduced sense of control over one’s action, will be familiar to anyone who has spoken, or received, an unkind word in anger. Society normally expects healthy adults to manage anger through a process of self-control, and many social institutions carefully teach such self-control. For example, anger management techniques teach individuals to “walk away before you lose it” (Graham 1998; Grave and Blissett 2004; Kendall 2000; NHS Choices 2015).

## Electronic supplementary material

Below is the link to the electronic supplementary material.

Supplementary material 1 (DOCX 34 KB)
