# Supplementary material for: I just lost it! Fear and anger reduce the sense of agency: a study using intentional binding
Source: Exp Brain Res. 2019 Mar 2;237(5):1205–12. doi: 10.1007/s00221-018-5461-6 (PMC6477810; doi:10.1007/s00221-018-5461-6)
Supplement: Supplementary file 1 — Supplementary material 1 (DOCX 34 KB) [file 221_2018_5461_MOESM1_ESM.docx]

**Supplementary material for:**

***I just lost it!* Fear and anger reduce sense of agency: a study using intentional binding**

Christensen, J.F., Di Costa, S., Beck, B., Haggard, P.

Institute of Cognitive Neuroscience, University College London (UCL)

1. **Methods**
   1. **Participants**

Group sizes for the two groups were determined with *GPower* 3.1. (Faul, Erdfelder, Lang, & Buchner, 2007) using the *a priori* procedure for dependent t-tests (based on: effect size = .80; alpha = .05; power = .95). The result was 19 participants; 20 were tested to allow full counterbalancing of conditions (threat block first vs. safe block first; Experiment 1).

A priori exclusion criteria were established as follows. In a post-session questionnaire participants in each experiment were asked how scared and how angry they felt (7-point Likert scale: -3 = not at all; +3 = very much). Participants in the fear experiments who indicated anger ratings over 0 were discarded (0 participants in experiment 1; 6 participants in experiment 2), as were participants in the anger experiment who indicated fear ratings of over 0 (6 participants, see below). Data from these participants was not analysed. Two participants in experiment 3, spontaneously reported a lack of interest in the experiment. They explicitly reported “I don’t care”, denying any motivation to adjust their performance to retain the bonus. These participants were judged as failing to comply with the instructions, and moreover being unwilling to comply. They were therefore also discarded without analysis, and replaced with two new participants. After exclusion and replacement, all 3 experiments had 20 participants. Data from discarded participants was not included in any analysis.

- 1. **Materials and Procedure**
     1. *Intentional binding task*

Participants were seated in front of a computer screen with an external SODIAL(R) Flexible Foldable USB keyboard in front of them to provide their responses on. At each trial, participants were instructed to press a key [ENTER] on this keyboard at the time of their choosing while fixating the centre of a clock displayed on the screen with a continuously rotating clock hand.

In all 3 experiments, participants made time estimations in the action binding task in 6 blocks of 32 trials. The first and last blocks were baseline blocks (i.e., no tone occurred). Blocks 2-5 were operant blocks, in which a tone ("beep") occurred 250 ms after the participant's keypress.

In each of these 4 operant blocks, 5 additional trials were added for the emotional state induction. In experiments on fear (i.e., experiments 1 and 2), of the blocks 2-5, two blocks were “threat” (fear) and two blocks were “safe” (control) blocks (fear experiments). In experiments on anger (i.e., experiment 3), these were “learning” (anger-induction) and “test” (control) blocks.

- - 1. *Fear induction*

The Threat of Shock paradigm was used for the induction of the laboratory analogue of fear. It is an extensively used paradigm in translational research on anxiety disorders to experimentally induce lab analogues of anxious states in healthy participants (Davis, Walker, Miles, & Grillon, 2010; Robinson, Vytal, Cornwell, & Grillon, 2013; Schmitz & Grillon, 2012). The key point of the paradigm is that participants anticipate a shock without actually receiving one. The procedure consists in delivering a couple of moderately painful shocks at the beginning of the target task and then run several blocks of the action binding task. Our participants performed 4 operant blocks of which 2 were “threat” blocks (the word “threat” was displayed on the screen) and participants were informed they could receive a shock at any time. The other 2 blocks were “safe” blocks (the word “safe” was displayed on the screen). Threat and Safe blocks were interleaved and counterbalanced (Experiment 1), or interleaved and fixed order (threat-safe-threat-safe; Experiment 2). In Experiment 1, the electric shocks occurred simultaneously with the key press. In experiment 2, they occurred at the time of the subsequent tone. This latter arrangement controls for potential effects of prior entry (subjective experience of salient events occurring earlier in time; Spence & Parise, 2010).

The effect of the fear induction is strongest when fewer shocks are actually being administered. Thus, in total participants received 5 shocks in each of the threat blocks. Before any analyses, the 5 shock trials in each of the 2 threat blocks were excluded, leaving 32 trials in each of the 4 operant blocks. In Experiment 2, participants also received an additional 5 low detection threshold pulses in each of the 2 safe blocks in order to equalise the number of events occurring in both the threat and the safe conditions. For this, the experimenter very visibly turned the shock level on the DS7 stimulator *down* to detection threshold after the threat block, and back *up* again to shock level after the safe block. The additional 5 trials happened on trials 2, 4, 7, 8, 30 (block 2, 1^st^ threat block); on trials 1, 9, 12, 13, 35 (block 3, 1^st^ safe block); on trials 3, 4, 7, 10, 16 (block 4, 2^nd^ threat block); and on trials 4, 11, 14, 17, 23 (block 5, 2^nd^ safe block). The location of the shock/pulse trials was specified (a) by previous literature specifying that the shocks should be in the beginning (Davis et al., 2010; Robinson et al., 2013; Schmitz & Grillon, 2012), and (b) by pilot experiment which confirmed the placement as adequate. The first pulse trial in the first safe block, however, was set to the first trial because pilot participants reported not to believe that the shocks in the safe block would be painless. By setting the first pulse to trial 1 instead of trial 7, we gave evidence that they need not fear shocks in the safe blocks.

For the pain calibration prior to Experiments 1 and 2 the shocks were delivered using a Digitimer DS7A constant current stimulator. Two electrodes were placed on the back of the participants’ non-dominant hand (left hand, as all participants were right handed). The individual pain threshold of each participant was determined using a step-wise approximation procedure, increasing the stimulation in small steps of 1 mA. During the calibration, the experimenter asked the participant a series of questions about the shocks, to find the level of shock which would be “painful, but definitely bearable”. The questions used were: “*is it uncomfortable?*”, ”*is it painful?”, “is it bearable?”, “could you cope with a maximum of 60 of these shocks?*”, “*could we increase the threshold?”* and finally, we would ask them “*on a scale from 0 to 10, where 0 is not painful at all and 10 is the worst possible pain; how painful is this shock?*”. Once the pain rating was “painful but definitely bearable”, the experimenter recorded the final rating (0 to 10) and this level was used as the participant’s individual painful shock.

In Experiment 1, in the pain calibration prior to the experimental task, participants had a mean detection threshold of 25 µv (SD = 5.13; range = 20 µv to 30 µv), a mean pain level of 225.75 µv (SD = 118.15; range = 60 µv to 480 µv) and a mean pain intensity rating (0; nothing to 10 worst possible) of 7.35 (SD = 0.99; range = 6 to 8.5). In the post-session questionnaire participants reported to have been fearful during the threat blocks (m = 1.15, SD = 1.35; range -2 to +3), and no participant reported to have been angry or annoyed during the experiment (m = -2.15, SD = 1.18; range: -3 to 0). Participants slightly overestimated the number of shocks they had received (correct answer: 10; estimated number: m = 10.25, SD = 3.19; range: 5 to 15). Most participants reported not being fearful or distressed after the experiment ended (How fearful are you right now: m = -1.65, SD = 1.39; range: -3 to +2).

In Experiment 2, participants had a mean detection threshold of 20.25 µv (SD = 4.17; range = 14 µv to 32 µv), a mean pain level of 178.25 µv (SD = 88.20; range = 40 µv to 430 µv) and a mean pain intensity rating (0; nothing to 10 worst possible) of 6.1 (SD = 2.21; range = 2 to 9). In the post-session questionnaire participants reported to have been fearful during the threat blocks (m = 0.8, SD = 1.77; range: -3 to +3), and not fearful in the safe blocks (m = -2.85; SD = 0.49; range: -1 to -3); (t = 2.09, df = 19, p < .001). Six participants reported anger ratings over 0 and were therefore discarded without analysis, and replaced by 6 new participants, according to pre-established exclusion criteria. The remaining participants reported no anger or annoyance during the experiment (m = -1.05, SD = 1.23; range: -3 to 0). Participants slightly overestimated the number of shocks they had received (correct answer: 10; estimated number: m = 10.2, SD = 3.41; range: 8 to 18). Most participants reported not being fearful or distressed after the experiment ended (How fearful are you right now: m = -0.7, SD = 1.38; range: -3 to +2).

- - 1. *Anger induction*

The Frustration or "Impossible Task" Paradigm is a common procedure for the induction of a lab analogue or an anger or frustration state. Examples are the Teacher-Learner paradigm (Buss, 1961), the Competitive RT game (Taylor, 1967), or the Hot sauce paradigm. In general, the mechanism these tasks rely on is that the solution of the assignment is never possible, no matter how hard the participant tries. Some of these paradigms additionally rely on the experimenter being abusive toward the participant. For example, in (Rohsenow & Bachorowski, 1984) participants were told draw a circle as slowly as possible. After this task was completed, an experimenter stated, "Obviously, you don't follow instructions”.

For the present experiment, participants were informed that in addition to the Clock estimation task, a RT task would be embedded in the 4 operant blocks. Whenever the screen background colour would change, they should press F1 as fast as possible using their right hand, crossing the body midline horizontally. They were told that in 2 “learning” blocks they would receive feedback on their performance (anger condition). If they would hear a beep sound that would mean that they had been fast enough. If they heard a “buzz” sound they had not been fast enough. They were promised a reward of £2.50 in addition to their final pay if they were fast enough in the RT trials. But to keep that bonus they would have to be fast enough. Every time they would hear a “buzz” sound this would mean that they had not been fast enough and they had lost 25p of the £2.50. In fact, the program was set thus that the outcome tone was always a buzz. Thus feedback was misleading, and frustrating for participants. Finally in the 2 remaining operant blocks, the “test” blocks (control condition), participants were informed that their RT would be monitored and they were to perform as fast as possible, just as they had learned in the “learning” blocks. Learning and test blocks were interleaved, fixed order, starting with a learning block.

The label ‘learning’ was chosen for the condition intended to produce high frustration and anger to give participants the impression that they could succeed and perform well if they made sufficient effort, coupled with actual feedback that they performed poorly. The label ‘test’ was chosen for the control condition and participants were informed that these blocks would be similar to the ‘learn’ blocks in terms of their task, but that these blocks were to ‘practice’ the task and that no feedback on their performance would be given in these blocks. The additional 5 trials happened on trials 2, 4, 7, 9, 30 (block 2, 1^st^ learning block); on trials 5, 9, 12, 14, 35 (block 3, 1^st^ test block); on trials 3, 5, 7, 10, 16 (block 4, 2^nd^ learning block); and on trials 4, 11, 14, 17, 23 (block 5, 2^nd^ test block). The rationale of making the trials slightly different in the anger experiment, as compared to the fear experiment was that in the fear experiment some of the shock trials were successive. Making trials successive also in the anger experiment would have made the task very difficult because of the reaching movement the participants had to perform.

Participants were faster in responding to the RT trials in the Anger blocks (m = 597ms; SE = 48ms), than in the Control blocks (m = 2297ms; SE = 159); t(19) = -11.25; p < .001). In the post-session questionnaire 6 participants reported fear ratings over 0, and were therefore discarded before analysis and replaced by 6 further participants, based on the pre-established exclusion criteria. The final set of participants reported to have been angry/frustrated during the anger blocks (m = 0.8, SD = 1.61; range: -3 to +3) and less angry in the control blocks (m = 0.1; SD = 1.65; range: -3 to +3); (t = 2.09, df = 19, p = .006), and were not fearful during the experiment (m = -2.1, SD = 1.21; range: -3 to 0). Participants slightly underestimated the number of RT trials they had received (correct answer: 10; estimated number in the anger blocks: m = 7.6, SD = 2.30; range: 4 to 13; estimated number in the control blocks: m = 6.4, SD = 2.35; range: 3 to 10). Most participants reported not being fearful or distressed after the experiment ended (How fearful are you right now: m = -2.45, SD = 1.10; range: -3 to +1).

Before any analyses, the 5 RT trials in each of the 2 anger blocks and the 2 control blocks were excluded, this left 32 trials in each of the 4 blocks.

To investigate whether attention was similar in experimental and control blocks, we conducted the same 2 x 2 RM ANOVA on the standard deviation of repeated timing judgements. No main effects or interactions were significant (Block: F(1,19) = .789, p = .073; State: F(1,19) = 2.126, p = .161; Block*State: F(1,19) = .280, p = .603).

- - 1. *Post session questionnaire*

In the post-session questionnaire participants were also asked to briefly write their impression about the experiment and they filled in the Behavioral inhibition and approach questionnaire (*BIS/BAS* scales; (Carver & White, 1994). See table 1 for participant data of the three experiments.

**Results**

- 1. **Experiment 1**

Participants exhibited less sense of agency in fearful compared to neutral conditions. A 2 x 2 RM ANOVA was conducted with the factors Occurrence (1^st^ time, 2^nd^ time) and State (Fear, Safe). It revealed a significant main effect of State (F(1,19) = 4.414, p = .049, η^2^ =.189). No other main effects or interactions were significant (Occurrence: F(1,19) = .353, p = .559, η^2^ =.018; Occurrence *State: F(1,19) = .619, p = .441, η^2^ =.032).

The same RM ANOVA was calculated again, adding the factor of Order as between subjects factor (threat vs safe block first). The main effect of State was again significant (F(1,19) = 5.007, p = .038, η^2^ =.218). There was a trend towards an interaction of State and Order (F(1,19) = 3.555, p = .076, η^2^ =.165). Simple effects t-tests were used to investigate the basis of this trend. When participants had the threat block first (then safe, then threat and safe again), the hypothesised effect was evident (t = -3.134, df = 9, p = .012). When participants had the safe block first (then threat, then safe and threat again), the effect was markedly reduced (t = -.234, df = 9, p = .820). This was taken into account for Experiment 2.

We additionally analysed the standard deviations of repeated time estimates, to check for possible changes in attention to the time judgement task. No main effects or interactions were significant (Occurrence: F(1,19) = .253, p = .253; State: F(19) = .001, p = .981; Occurrence *State: F(1) = .096, p = .760).

- 1. **Experiment 2**

Experiment 2 replicated experiment 1, with some methodological adjustments. The *Threat of Shock* paradigm was again used to induce a fearful state in the participants. We now presented shocks at the time of the tone to avoid the possibility that the shock altered time estimation by providing an additional salient, attention-capturing event, independent of action and outcome (‘prior entry’; (Spence & Parise, 2010). In Experiment 1 the shock was located at the time of the key press (see Materials). Second, we introduced a number of non-painful shocks in the safe blocks, so that the number of trials was balanced across conditions. These shocks were at the participant’s detection threshold level, as established by a staircase procedure with 3 reversals. Third, the block order was fixed, to always begin with threat. Finally, an additional question in the post session questionnaire was included, asking how scared they had felt in the safe blocks (to contrast it with their ratings in the threat blocks).

A 2 x 2 Repeated measures (RM) ANOVA was conducted with the factors Occurrence (1^st^ time, 2^nd^ time) and State (Fear, Safe). As predicted, there was a clear trend of State (F(1,19) = 3.836, p = .065, η^2^ =.168), in the same direction as experiment 1: participants showed a trend towards less action binding in a fear state. No other main effects or interactions were significant (Occurrence: F(1,19) = .310, p = .584, η^2^ =.016; Occurrence *State: F(1,19) = 1.308, p = .267, η^2^ =.064).

To investigate whether attention was similar in experimental and control blocks, we conducted the same 2 x 2 RM ANOVA on the standard deviation of repeated timing judgements. No main effects or interactions were significant (Occurrence: F(1,19) = .258, p = .914; State: F(1,19) = .668, p = .424; Occurrence *State: F(1,19) = .258, p = .618).

- 1. **Experiment 3**

A 2 x 2 Repeated measures (RM) ANOVA was conducted with the factors Occurrence (1^st^ time, 2^nd^ time) and State (Anger, Control). It revealed a significant main effect of State (F(1,19) = 4.847, p = .040, η^2^ =.203). Participants showed reduced action binding when angry, contrary to our predictions. No other main effects or interactions were significant (Occurrence: F(1,19) = .385, p = .542, η^2^ =.020; Occurrence *State: F(1,19) = 1.860, p = .189, η^2^ =.089).

1. **Relevance to legal concepts of responsibility**

Our investigation was specifically designed to provide a neuropsychological model relevant to the cognitive mechanisms underlying the sociolegal concept of *loss of control*. Several legal systems, such as that in the UK (Coroners and Justice Act, 2009; article 54, sect. 1a), can partly or wholly exempt the agent from legal punishment, where the loss of control is due to a qualifying trigger or provocation associated with strong emotion, such as fear or anger. Taken at face value, this allowance suggests that strong emotion partly softens the normal legal view of the individual as a conscious, voluntary agent, aware of both their actions and consequences, and choosing to initiate actions on the basis of reasoned knowledge about actions, agents and consequences.

Perhaps unsurprisingly, this defence has proved deeply controversial for several reasons. First, courts have, in general, interpreted the voluntary act condition in an absolute way (Hart & Honore, 1985), and have viewed situational factors as having only minor implications for responsibility. In essence, few psychological features are as fundamental to human nature as the ability to control one’s own actions in an appropriate way. Second, legislators, academics and jurisdictions disagree on exactly which emotions may lead to a loss of control of action, and on how or why the emotion might then justify the action. *Crime passionel* offers one hotly-debated example: the most recent UK law specifically ruled out sexual infidelity as grounds for loss of control/diminished responsibility (Coroners and Justice Act, 2009; article 54, sect. 6c), yet this defence remains available in other jurisdictions (e.g., in Uruguay; Uruguay Penal Code, article 36). This debate has been limited by the lack of clear evidence for a relation between emotional state and responsibility. Here, we sought to investigate how the agent’s emotional state of fear or anger may affect their experience of having caused the outcomes of their actions. We found a reduced perceptual association between action and outcome. Our experimental findings are broadly consistent with the legal concept that strong emotion can reduce sense of agency.

**References**

Buss, A. (1961). *Investigating Aggression in the Laboratory. The psychology of aggression*. Hoboken, NJ, US: : John Wiley & Sons Inc.

Coroners and Justice Act 2009; article 54. https://www.legislation.gov.uk/ukpga/2009/25/section/55

Carver, C. S., & White, T. L. (1994). Behavioral inhibition, behavioral activation, and affective responses to impending reward and punishment: The BIS/BAS scales. *Journal of Personality and Social Psychology, 67*, 319-333.

Davis, M., Walker, D. L., Miles, L., & Grillon, C. (2010). Phasic vs sustained fear in rats and humans: role of the extended amygdala in fear vs anxiety. *Neuropsychopharmacology, 35*, 105-135.

Faul, F., Erdfelder, E., Lang, A.-G., & Buchner, A. (2007). G*Power 3: A flexible statistical power analysis program for the social, behavioral, and biomedical sciences. *Behavior Research Methods, 39*, 175-191.

Robinson, O. J., Vytal, K., Cornwell, B. R., & Grillon, C. (2013). The impact of anxiety upon cognition: perspectives from human threat of shock studies. *Frontiers in Human Neuroscience, 7*, 203.

Rohsenow, D. J., & Bachorowski, J. (1984). Effect of alcohol and expectancies on verbal aggression in men and women. *Journal of Abnormal Psychology, 93*, 418-432.

Schmitz, A., & Grillon, C. (2012). Assessing fear and anxiety in humans using the threat of predictable and unpredictable aversive events (the NPU-threat test). *Nature Protocols, 7*, 527-532.

Spence, C., & Parise, C. (2010). Prior-entry: A review. *Consciousness and Cognition, 19*(1), 364-379.

Taylor, S. P. (1967). Aggressive behavior and physiological arousal as a function of provocation and the tendency to inhibit aggression. *Journal of Personality, 35*, 297-310. doi:10.1111/j.1467-6494.1967.tb01430.x
